# Parkinson’s Disease Patients Face Higher 90-Day Readmission, Reoperation, and Infection Risk Following Total Knee Arthroplasty

**DOI:** 10.1016/j.artd.2026.101970

**Published:** 2026-02-28

**Authors:** David Maman, Yaniv Steinfeld, Yaron Berkovich

**Affiliations:** a Department of Orthopaedic Surgery, Carmel Medical Center, Haifa, Israel; b Faculty of Medicine, Technion – Israel Institute of Technology, Haifa, Israel

**Keywords:** Total knee arthroplasty, Parkinson’s disease, Readmission, Complications, Length of stay, National Readmission Database

## Abstract

**Background:**

Parkinson’s disease (PD) is an increasingly common comorbidity in patients undergoing total knee arthroplasty (TKA), yet its impact on readmissions remains poorly defined.

**Methods:**

Using the Nationwide Readmissions Database 2020-2022, we identified elective, primary TKA cases. Patients with oncologic diagnoses, COVID-19-related admissions, acute fractures, and contralateral knee arthroplasty were excluded. Only the first readmission within 90 days was analyzed. Propensity score matching (1:1) was performed for demographics, comorbidities, hospital factors, and primary payer. The primary outcome was 90-day readmission. Secondary outcomes included infection-related readmissions, surgical procedures during readmission, readmission length of stay, readmission costs, and in-hospital mortality.

**Results:**

After matching, 7286 patients were included (3675 PD vs 3611 non-PD). PD patients had a significantly higher 90-day readmission rate (13.0% vs 7.3%; *P* < .01). Infection-related readmissions (4.1% vs 2.2%; *P* < .01) and surgical procedures during readmission (7.5% vs 4.1%; *P* < .01) were more frequent in PD patients. Readmission length of stay was longer (5.9 vs 4.1 days; *P* < .01), and readmission costs were higher ($63,023 vs $48,855; *P* < .01). In-hospital mortality during readmission was also higher in PD patients (0.3% vs 0.1%; *P* < .01).

**Conclusions:**

PD is associated with a significantly higher risk of 90-day readmission following TKA, with readmissions frequently involving infection-related complications and surgical intervention. These findings highlight the importance of tailored perioperative care and enhanced postdischarge monitoring in this vulnerable population.

**Levels of Evidence:**

Level III.

## Introduction

Total knee arthroplasty (TKA) is among the most frequently performed orthopaedic procedures worldwide, with over 1 million cases projected annually in the United States [1]. Despite advances in perioperative management, hospital readmission after TKA remains an important quality and cost metric, influencing bundled payment models and patient outcome [2,3].

Parkinson’s disease (PD) is a progressive neurodegenerative disorder characterized by tremor, rigidity, postural instability, and autonomic dysfunction [4]. With an aging population, PD is increasingly encountered among arthroplasty patients [5]. Prior studies have suggested higher complication rates in PD, including periprosthetic joint infection and impaired functional recovery, but robust nationwide data on readmission risk remain relatively sparse [[6], [7], [8]].

Smaller, single-institution and database studies have hinted at increased postoperative complications in PD patients undergoing joint replacement [[6], [7], [8]], and limited large-scale claims analyses suggest elevated 90-day readmission and costs after TKA in PD [[9], [10], [11]].

The purpose of this study was to evaluate the association between PD and 90-day readmission after TKA using a large national dataset and propensity score matching. We hypothesized that PD patients would experience higher readmission rates, increased infection-related complications, greater surgical intervention during readmission, longer hospital stays, higher costs, and increased mortality compared with non-PD patients.

## Material and methods

### Data source

We used the Healthcare Cost and Utilization Project (HCUP) Nationwide Readmissions Database (NRD) for the years 2020-2022. The NRD provides nationally representative data on hospital discharges across the United States and enables longitudinal tracking of readmissions within the same calendar year.

### Cohort selection

Patients undergoing elective primary TKA were identified using International Classification of Diseases (ICD)-10-Procedure Coding System procedure codes in the primary procedure field (I10_PR1):

0SRC069, 0SRC06A, 0SRC06Z, 0SRC07Z, 0SRC0J9, 0SRC0JA, 0SRC0JZ, 0SRC0KZ, 0SRC0L9, 0SRC0LA, 0SRC0LZ, 0SRD069, 0SRD06A, 0SRD06Z, 0SRD07Z, 0SRD0J9, 0SRD0JA, 0SRD0JZ, 0SRD0KZ, 0SRD0L9, 0SRD0LA, 0SRD0LZ.

Robotic-assisted cases were identified using ICD-10-Procedure Coding System codes 8E0Y0CZ and 8E0YXCZ.

### Exclusion criteria included

• Nonelective admissions.
• Revision TKA at the index admission.
• Bilateral procedures.
• Patients <18 years.
• Oncology-related, fracture-related, or reoperation admissions.
• Any case coded for COVID-19 (U07.1).
• Index discharges after September of each year (to ensure complete 90-day follow-up).
• Patients with missing demographic or hospital identifiers.

For patients with multiple readmissions, only the first readmission within 90 days of discharge was analyzed to avoid double-counting.

### Exposure variable

The exposure of interest was a diagnosis of PD (ICD-10-Clinical Modification G20.x) at the index admission.

### Outcomes

The primary outcome was 90-day hospital readmission.

Secondary outcomes included:

• Infection-related readmissions: periprosthetic joint infection, wound dehiscence, sepsis, cellulitis/soft tissue infection, pneumonia, urinary tract infection.
• Performance of any surgical procedure during readmission.
• Readmission length of stay (LOS).
• Readmission hospital charges, converted to costs using HCUP cost-to-charge ratios.
• In-hospital mortality during readmission.

### Statistical analysis

We performed 1:1 propensity score matching (nearest neighbor, caliper 0.1, without replacement) between PD and non-PD patients. Matching variables included age, sex, primary payer, hospital region, hospital teaching status, and comorbidities (hypertension, diabetes, obesity, chronic obstructive pulmonary disease, chronic kidney disease, congestive heart failure, dyslipidemia, anemia, osteoporosis, thyroid disease, alcohol abuse, and obstructive sleep apnea).

Balance was assessed with standardized mean differences, with <0.1 considered acceptable. Categorical variables were compared using χ^2^ tests, and continuous variables using *t*-tests or Wilcoxon rank-sum tests, as appropriate. Kaplan-Meier survival analysis with log-rank testing was performed to compare 90-day readmission-free survival between groups. A 2-sided *P* value <.05 was considered statistically significant. Analyses were conducted using SPSS (IBM Corp, Armonk, NY).

### Ethical considerations

The NRD contains deidentified, publicly available data, and therefore this study was exempt from institutional review board approval and informed consent requirements, in accordance with the U.S. Department of Health and Human Services regulations (45 CFR 46.101[b]). Data use followed the HCUP Data Use Agreement.

## Results

### Baseline characteristics

A total of 483,762 patients who underwent elective primary TKA between 2020 and 2022 were included, of whom 3675 (0.8%) had a diagnosis of PD (Table 1). Compared with non-PD patients, those with PD were significantly older at the time of surgery (72.3 vs 67.8 years; *P* < .01) and less frequently female (50.1% vs 61.8%; *P* < .01). Several comorbidities were more prevalent in the PD cohort, including dyslipidemia (54.7% vs 51.6%; *P* < .01), obstructive sleep apnea (17.6% vs 16.0%; *P* = .01), chronic anemia (7.0% vs 5.9%; *P* = .01), osteoporosis (7.0% vs 4.6%; *P* < .01), chronic kidney disease (13.6% vs 9.7%; *P* < .01), congestive heart failure (2.2% vs 1.5%; *P* < .01), and chronic lung disease (7.3% vs 6.3%; *P* = .022). Conversely, obesity was significantly less common among PD patients (25.7% vs 35.7%; *P* < .01).

**Table 1:** Baseline characteristics of patients undergoing primary total knee arthroplasty (NRD 2020-2022), stratified by Parkinson’s disease diagnosis.

Characteristic	Non-PD	PD	*P* value
Age, years (mean ± SD)	67.8 ± 9.6	72.3 ± 7.7	<.01
Calendar year (mean ± SD)	2020.8 ± 0.8	2020.9 ± 0.8	<.01
Female (%)	61.8	50.1	<.01
Robotic-assisted TKA (%)	10	11.1	.03
Dyslipidemia (%)	51.6	54.7	<.01
Obstructive sleep apnea (%)	16	17.6	.01
Chronic anemia (%)	5.9	7	.01
Alcohol abuse (%)	0.9	0.7	.21
Osteoporosis (%)	4.6	7	<.01
Type 2 diabetes (%)	22.6	21.6	.16
Chronic kidney disease (%)	9.7	13.6	<.01
Congestive heart failure (%)	1.5	2.2	<.01
Chronic lung disease (%)	6.3	7.3	.022
Thyroid disorder (%)	18.2	19.2	.11
Liver disease (%)	1.9	1.9	.97
Obesity (%)	35.7	25.7	<.01

### Baseline characteristics after propensity score matching

After propensity score matching, we obtained 3675 patients with PD and 3611 matched non-PD controls, resulting in a well-balanced cohort of 7286 patients (Table 2). Baseline demographic and clinical characteristics were comparable between groups, with no statistically significant differences observed in age, sex distribution, or comorbidities. This indicates successful covariate balance following matching.

**Table 2:** Baseline characteristics after 1:1 propensity score matching between patients with and without Parkinson’s disease undergoing elective primary TKA (n = 7286).

Characteristic	Non-PD	PD	*P* value
Age, years (mean ± SD)	72.34 ± 7.67	72.33 ± 7.67	.97
Calendar year (mean ± SD)	2020.87 ± 0.82	2020.87 ± 0.81	.69
Female (%)	50.3	50.1	.69
Robotic-assisted TKA (%)	10.9	11.1	.74
Dyslipidemia (%)	55.7	54.7	.37
Obstructive sleep apnea (%)	17	17.6	.5
Chronic anemia (%)	7.4	7	.51
Alcohol abuse (%)	0.6	0.7	.51
Osteoporosis (%)	7.6	7	.33
Type 2 diabetes (%)	21.5	21.6	.9
Chronic kidney disease (%)	13.2	13.6	.67
Congestive heart failure (%)	2	2.2	.65
Chronic lung disease (%)	7.2	7.3	.95
Thyroid disorder (%)	2.1	1.9	.54
Liver disease (%)	18.4	19.2	.39
Obesity (%)	25.9	25.7	.82

### Postoperative outcomes following elective TKA

Table 3 summarizes outcomes during the index hospitalization and within 90 days of discharge. Compared with matched non-PD patients, those with PD experienced a significantly longer index hospital stay (3.01 vs 2.19 days; *P* < .01), although index hospitalization costs were comparable between groups.

**Table 3:** Index hospitalization and 90-day outcomes after matching (PD vs non-PD).

Outcome	Non-PD (n = 3611)	PD (n = 3675)	*P* value
Index hospitalization			
Index LOS, days (mean ± SD)	2.19 ± 2.32	3.01 ± 3.50	<.01
Index cost, $ (mean ± SD)	73,863.84 ± 48,127.60	75,797.73 ± 52,328.62	.1
Ninety-day outcomes			
All-cause readmission (%)	7.3	13	<.01
Infection-related readmission (%)	2.2	4.4	<.01
Any surgery during readmission (%)	4.1	7.5	<.01
Readmission LOS, days (mean ± SD)	4.08 ± 3.68	5.88 ± 5.11	<.01
Readmission cost, $ (mean ± SD)	48,855.30 ± 48,754.06	63,023.53 ± 65,516.53	<.01
In-hospital mortality during readmission (%)	0.1	0.3	<.01

Within 90 days, patients with PD had a markedly higher rate of all-cause readmission (13.0% vs 7.3%; *P* < .01). Infection-related readmissions were more frequent among PD patients (4.1% vs 2.2%; *P* < .01). Surgical procedures during readmission were also more common in PD (7.5% vs 4.1%; *P* < .01).

Readmissions in PD patients were associated with significantly longer hospital stays (5.88 vs 4.08 days; *P* < .01) and higher costs ($63,023 vs $48,855; *P* < .01). Mortality during readmission, although rare, was 3 times higher in PD patients (0.3% vs 0.1%; *P* < .01).

### Infection-related readmissions

We next examined the specific causes of infection-related readmissions (Table 4). Patients with PD showed a consistently higher burden of serious postoperative infections compared with non-PD controls. Notably, the incidence of periprosthetic joint infection (1.1% vs 0.5%; *P* < .01) and sepsis/bacteremia (0.8% vs 0.1%) was markedly increased in the PD cohort. Although cellulitis/soft tissue infections were slightly more common in non-PD patients (1.3% vs 0.5%), the aggregate burden of infection-related readmissions was significantly greater in PD patients (4.1% vs 2.2%; *P* < .01).

**Table 4:** Specific infection-related causes of 90-day readmission in patients with and without Parkinson’s disease undergoing elective TKA.

Infection-related cause (%)	Non-PD	PD	*P* value
Periprosthetic joint infection	0.5	1.1	<.01
Wound dehiscence	0.1	1	
Sepsis/bacteremia	0.1	0.8	
Cellulitis/soft tissue infection	1.3	0.5	
Respiratory infection/pneumonia	0.1	0.3	
Urinary tract infection/pyelonephritis	0.1	0.4	
Any infection-related readmission	**2.2**	4.1	

### Kaplan-Meier survival analysis

**Figure 1:**
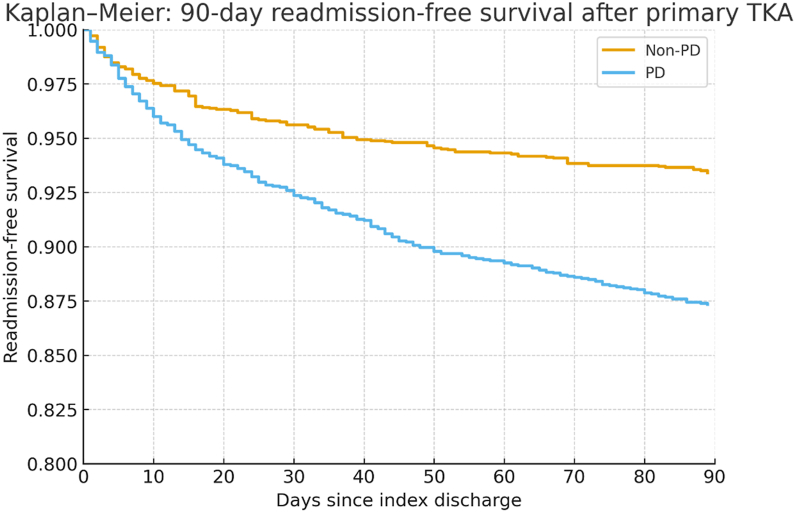
Kaplan-Meier curves demonstrating 90-day readmission-free survival following elective total knee arthroplasty (TKA) in patients with and without Parkinson’s disease.

We further examined temporal patterns of readmission using Kaplan-Meier survival curves (Fig. 1). Readmission-free survival within 90 days after elective TKA was significantly lower in patients with PD compared with matched non-PD controls. Divergence between groups emerged early in the postoperative period and continued to widen over time.

On log-rank testing, the difference in 90-day readmission-free survival between PD and non-PD patients was statistically significant (*P* < .01).

## Discussion

This nationwide, propensity-matched analysis demonstrates that PD is associated with nearly double the risk of 90-day readmission after elective TKA, consistent with prior national database analyses reporting higher complication and cost burdens in PD following joint arthroplasty [12]. Infection-related complications, surgical interventions, and readmission mortality were also higher among PD patients, aligning with recent multicenter and registry-based reports [7,8,13].

Our findings extended earlier single-institution and small cohort studies that suggested increased complications and functional decline in PD but lacked statistical power for national readmission outcomes [7,8,14]. By analyzing >7000 matched patients, the present study is, to our knowledge, among the first to quantify nationwide 90-day readmission burden and infection-driven readmissions after TKA in PD [7,12,13].

Although we used propensity score matching to balance measured demographics, comorbidities, payer, and hospital characteristics, this retrospective analysis of administrative data supports association rather than causation. Propensity matching can reduce confounding by measured variables but cannot address residual confounding from unmeasured factors (eg, functional status, frailty, cognitive impairment, nutritional status, caregiver support, medication adherence, and PD severity). Therefore, our findings should be interpreted as PD being a marker of higher postoperative vulnerability and resource utilization rather than proof that PD itself directly causes readmission events.

Several mechanisms may explain these observations. PD-related motor symptoms (bradykinesia, rigidity, tremor, postural instability) can delay mobilization and impair wound healing; recent data confirm higher postoperative complication risk across surgical cohorts [15]. Nonmotor features are also critical: dysphagia is common and independently predicts aspiration pneumonia, prolonged hospitalization, and mortality [16,17]. Autonomic dysfunction manifesting as orthostatic hypotension, urinary retention, and gastrointestinal dysmotility further predisposes to hemodynamic instability, urinary tract infection, and impaired tissue repair [18]. Reviews of perioperative management highlight pulmonary aspiration, urinary tract infection, wound infection, and respiratory insufficiency as the leading complications in PD surgery [19]. Sarcopenia, prevalent in PD, compounds frailty and infection risk. [20] Together, these motor and nonmotor features plausibly increase susceptibility to postoperative infection, delay recovery, prolong hospitalization, and magnify the impact of complications, thereby contributing to the higher readmission rates, longer LOS, and increased in-hospital mortality observed in patients with PD.

Clinically, these findings emphasize the importance of perioperative optimization, dysphagia screening, infection-prevention bundles, orthostatic-hypotension management, and tailored discharge planning. [19,21] Specific measures may include time-critical continuation of dopaminergic therapy, avoidance of dopamine-antagonist antiemetics, early swallow assessment in high-risk patients, enhanced pulmonary hygiene, and intensified postdischarge follow-up, in accordance with published perioperative PD management guidelines.

Economically, PD patients had longer readmission LOS and higher costs, paralleling prior evidence that PD increases episode-of-care spending after TKA [3,12]. Within bundled payment frameworks such as Medicare’s Comprehensive Care for Joint Replacement model, identifying high-risk populations like PD can guide resource allocation and targeted care pathways [2]. However, readmission LOS and costs are also influenced by hospital practice patterns, discharge destination availability, and overall patient complexity, and should therefore be interpreted as episode-of-care resource utilization associated with PD rather than a direct cost effect of the disease itself.

This study has several limitations inherent to its retrospective design and use of administrative claims data. First, although propensity score matching was employed to balance measured demographics, comorbidities, payer, and hospital characteristics, residual confounding from unmeasured factors remains possible [22,23]. Variables such as functional status, frailty, cognitive impairment, socioeconomic factors, caregiver support, and medication adherence are not captured in the NRD and may contribute to postoperative risk, limiting causal inference. Accordingly, our findings should be interpreted as demonstrating an association rather than a causal relationship between PD and adverse post-TKA outcomes. Although PD was present at the index hospitalization and therefore preceded postoperative complications and readmissions, these outcomes may also reflect interacting comorbidities and patient complexity rather than effects attributable to PD alone.

Second, identification of PD relied on ICD-10-Clinical Modification diagnostic coding, which is subject to undercoding, miscoding, and interinstitutional variability. Although prior validation studies support the use of administrative codes for identifying PD, some degree of misclassification is unavoidable and may bias effect estimates toward or away from the null. In addition, the NRD captures inpatient hospitalizations only; therefore, our cohort reflects patients undergoing elective inpatient primary TKA within participating states. Exclusion of index discharges after September to ensure complete 90-day follow-up, while methodologically necessary, may further limit generalizability to outpatient or ambulatory surgery center arthroplasty pathways.

Third, the NRD lacks granular clinical detail regarding PD severity (eg, Hoehn and Yahr stage), motor fluctuation burden, cognitive status, and outpatient medication regimens, including perioperative timing of dopaminergic therapy. These factors may meaningfully influence postoperative mobility, aspiration risk, autonomic instability, delirium, and infection susceptibility. Their absence precludes stratified risk assessment by disease severity and may introduce bias if more advanced PD is overrepresented among readmitted patients. Consequently, the reported estimates represent an average effect across coded PD diagnoses, rather than severity-specific risk. Future studies incorporating clinical staging, medication timing, and functional measures are needed to further clarify mechanisms and identify modifiable targets for risk reduction.

Despite these limitations, the study’s strengths include a large, contemporary, nationally representative sample, rigorous exclusion criteria, and robust propensity-matched design, supporting the validity and relevance of the observed associations.

## Conclusions

Patients with PD undergoing elective primary TKA are at substantially increased risk of 90-day readmission, particularly due to infection-related complications, and experience longer and more costly hospitalizations, as well as higher in-hospital mortality during readmission. These findings underscore the importance of recognizing PD as a high-risk condition in the context of elective arthroplasty. Surgeons should incorporate this risk into preoperative counseling and optimization strategies, while health systems should consider tailored pathways for perioperative management and discharge planning. Further studies are warranted to explore strategies to reduce postoperative complications and improve outcomes in this vulnerable patient population.

## Ethical approval

The study was conducted using deidentified HCUP NRD data and was exempt from institutional review board approval.

## Conflicts of interest

The authors declare there are no conflicts of interest.

For full disclosure statements refer to https://doi.org/10.1016/j.artd.2026.101970.

## CRediT authorship contribution statement

**David Maman:** Writing – original draft, Validation, Methodology, Formal analysis, Conceptualization. **Yaniv Steinfeld:** Supervision, Project administration. **Yaron Berkovich:** Supervision, Project administration, Conceptualization.

## Figures and Tables

**PD 90 days:**
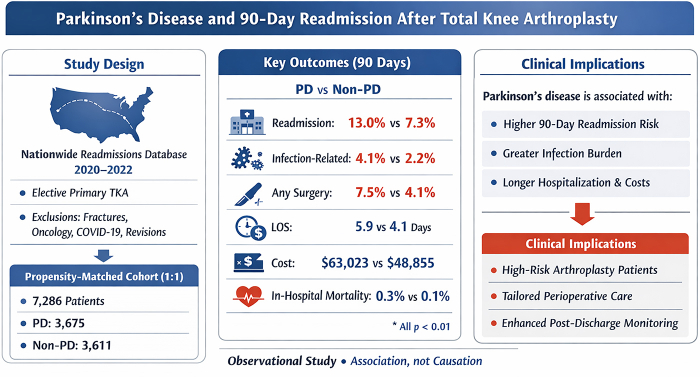


## Appendix A Supplementary data

Conflict of Interest Statement for Berkovich

Conflict of Interest Statement for Maman

Conflict of Interest Statement for Steinfeld

Highlights
