# Supplementary material for: Parkinson’s Disease Patients Face Higher 90-Day Readmission, Reoperation, and Infection Risk Following Total Knee Arthroplasty
Source: Arthroplast Today. 2026 Feb 28;38:101970. doi: 10.1016/j.artd.2026.101970 (PMC12966648; doi:10.1016/j.artd.2026.101970)
Supplement: Conflict of Interest Statement for Maman [file mmc2.docx]

# CONFLICT OF INTEREST STATEMENT

American Association of Hip and Knee Surgeons
(Adopted from the American Academy of Orthopaedic Surgeons disclosure statement)

Manuscript Title:
Parkinson’s Disease Patients Face Higher 90-Day Readmission, Reoperation, and Infection Risk Following TKA

1. Royalties from a company or supplier — None.

2. Speakers bureau/paid presentations for a company or supplier — None.

3A. Paid employee for a company or supplier — None.

3B. Paid consultant for a company or supplier — None.

3C. Unpaid consultant for a company or supplier — None.

4. Stock or stock options in a company or supplier — None.

5. Research support from a company or supplier as a Principal Investigator — None.

6. Other financial or material support from a company or supplier — None.

7. Royalties, financial or material support from publishers — None.

8. Medical/Orthopaedic publications editorial/governing board — None.

9. Board member/committee appointments for a society — None.

Author Name (Print or Type): David Maman, MD

Author Signature: _____________________________

Date: ___________25.10.2025__________________
