# Supplementary material for: Parkinson’s Disease Patients Face Higher 90-Day Readmission, Reoperation, and Infection Risk Following Total Knee Arthroplasty
Source: Arthroplast Today. 2026 Feb 28;38:101970. doi: 10.1016/j.artd.2026.101970 (PMC12966648; doi:10.1016/j.artd.2026.101970)
Supplement: Highlights [file mmc4.docx]

**Highlights**

- Parkinson’s disease is associated with higher 90-day readmission after TKA.
- PD patients have higher infection-related readmissions and reintervention rates.
- Readmission length of stay and costs are higher in patients with PD.
- Findings support PD-specific risk stratification and perioperative pathways.
